# The *ERECTA*, *CLAVATA* and class *III HD-ZIP* Pathways Display Synergistic Interactions in Regulating Floral Meristem Activities

**DOI:** 10.1371/journal.pone.0125408

**Published:** 2015-05-06

**Authors:** Udi Landau, Lior Asis, Leor Eshed Williams

**Affiliations:** The Robert H. Smith Institute of Plant Sciences & Genetics in Agriculture, The Robert H. Smith Faculty of Agriculture, Food and Environment, The Hebrew University of Jerusalem, Rehovot, Israel; Ecole Normale Superieure, FRANCE

## Abstract

In angiosperms, the production of flowers marks the beginning of the reproductive phase. At the emergence of flower primordia on the flanks of the inflorescence meristem, the *WUSCHEL (WUS)* gene, which encodes a homeodomain transcription factor starts to be expressed and establishes *de novo* stem cell population, founder of the floral meristem (FM). Similarly to the shoot apical meristem a precise spatial and temporal expression pattern of *WUS* is required and maintained through strict regulation by multiple regulatory inputs to maintain stem cell homeostasis. However, following the formation of a genetically determined fixed number of floral organs, this homeostasis is shifted towards organogenesis and the FM is terminated. In here we performed a genetic study to test how a reduction in *ERECTA*, *CLAVATA* and *class III HD-ZIP* pathways affects floral meristem activity and flower development. We revealed strong synergistic phenotypes of extra flower number, supernumerary whorls, total loss of determinacy and extreme enlargement of the meristem as compared to any double mutant combination indicating that the three pathways, *CLV3*, *ER* and *HD-ZIPIII* distinctively regulate meristem activity and that they act in parallel. Our findings yield several new insights into stem cell-driven development. We demonstrate the crucial requirement for coupling floral meristem termination with carpel formation to ensure successful reproduction in plants. We also show how regulation of meristem size and alternation in spatial structure of the meristem serve as a mechanism to determine flower organogenesis. We propose that the loss of FM determinacy due to the reduction in CLV3, ER and HD-ZIPIII activity is genetically separable from the *AGAMOUS* core mechanism of meristem termination.

## Introduction

In angiosperms, the production of flowers marks the beginning of the reproductive phase. At the emergence of flower primordia on the flanks of the inflorescence meristem, the *WUSCHEL* (*WUS*) gene, which encodes a homeodomain transcription factor, starts to be expressed in a few cells known as the organizing center (OC). WUS then specifies stem cell identity in the overlying cells to establish *de novo* stem cell population, founder of the floral meristem (FM) [1–3]. The stem cells divide and their daughter cells can either remain stem cells or proliferate before being incorporated into floral organ primordia. To maintain the organization of the FM, an homeostasis which is the balance between stem cell renewal, cell proliferation and cell differentiation, must be kept [4]. Similar to the shoot apical meristem (SAM), stem cell homeostasis within the FM is mediated by the CLAVATA–WUS feedback loop [5–7]. However, following the formation of a genetically determined fixed number of floral organs, this homeostasis is shifted towards organogenesis and the FM activity terminates. Genetic studies have identified numerous mutants in which the homeostasis between stem cell population size and cells that are recruited for floral organ primordia formation is disrupted, leading to a decrease or increase in floral organ number. For example mutations in *WUS*, *TOUSLED* and *AUXIN RESISTANT 6* genes lead to reduced meristem size and organ number [1,8,9], whereas loss-of-function alleles of *CLAVATA3* (*CLV3*), *ULTRAPETALA1* (*ULT1*) and *PLURIPETATA* lead to an increase in FM size and floral organ number [5,6,10,11].

In *Arabidopsis thaliana* the flowers consist of four sepals, four petals, six stamens and a bi-carpellate gynoecium arising in four concentric whorls in a centripetal chronological manner (i.e. with the outer whorls developing before the inner ones).

During early flower development, the flower primordium becomes separated from the inflorescence meristem and starts to grow larger very quickly in all directions to increase meristem size and to provide sufficient cells for the formation of floral organ primordia [12]. The sepal primordia appear first concurrently with further increase in the diameter of the developing flower primordium [13]. Next the petal and stamen primordia become visible. At floral stage 6 (stages according to Smyth *et al*. [13]), simultaneously with FM termination a rim at the central dome of the flower primordium grows upward to produce a hollow tube that will become the gynoecium consisting of two fused ovule-bearing carpels [14,15]. Early in flower development, at floral stage 3, WUS activates the expression of the *AGAMOUS* (*AG*) MADS-box gene, a central factor in specifying stamen and carpel identities [16]. AG in turn terminates floral stem cell maintenance by repressing *WUS* expression thus promoting meristem determinacy [2,17–19]. Mutations in *AG* cause the formation of indeterminate meristem which produces flowers-within-flower phenotype with no stamens and carpels [20–22]. Accordingly, late stage *ag* mutant flower still shows strong *WUS* expression in the center of a regular size floral meristem [17].

Therefore, to allow proper flower development, a precise spatial and temporal expression pattern of *WUS* is required and maintained through strict regulation by multiple regulatory inputs.

It was shown by several independent groups that the ERECTA receptor like kinase (ER), the CLAVATA (CLV) signaling pathway and class III HD ZIP (HD-ZIPIII) factors regulate SAM size via regulation of *WUS* expression [23–32].

Our recent study demonstrated that all three act in separate pathways at the SAM and have similar functions in the FM [33]. Here, we further address how loss-of-function in those pathways affects FM activity and flower development, and demonstrates the importance of cell proliferation regulation for correct flower organogenesis to ensure successful reproduction.

## Results and Discussion

### The *ERECTA*, *CLAVATA* and class III *HD-ZIP* pathways synergistically regulate floral meristem activity

To investigate the interaction of the *ER*, *CLV* and *HD-ZIPIII* pathways in regulating floral meristem activity, we analyzed flowers of single, double and triple mutant combinations of *clv3-2*, *er* and *jabba-1D* (*jba-1D*). In the *jba-1D* mutant overexpression of *miR166g* leads to decrease in several *HD-ZIPIII* transcripts level leading to up-regulation of *WUS* expression and enlargement of the SAM [25]. No detailed analysis of FM size or floral organ numbers has been reported for *jba-1D*. Loss of function of the three *ER* family genes leads to flattened and broadened SAM and aberrant flower development [34].Yet the single mutant does not exhibit increase in FM size or in floral organ number. However, the *clv3-2* mutant exhibits enlarged floral meristem and increased number of floral organs [5,6].

**Fig 1:**
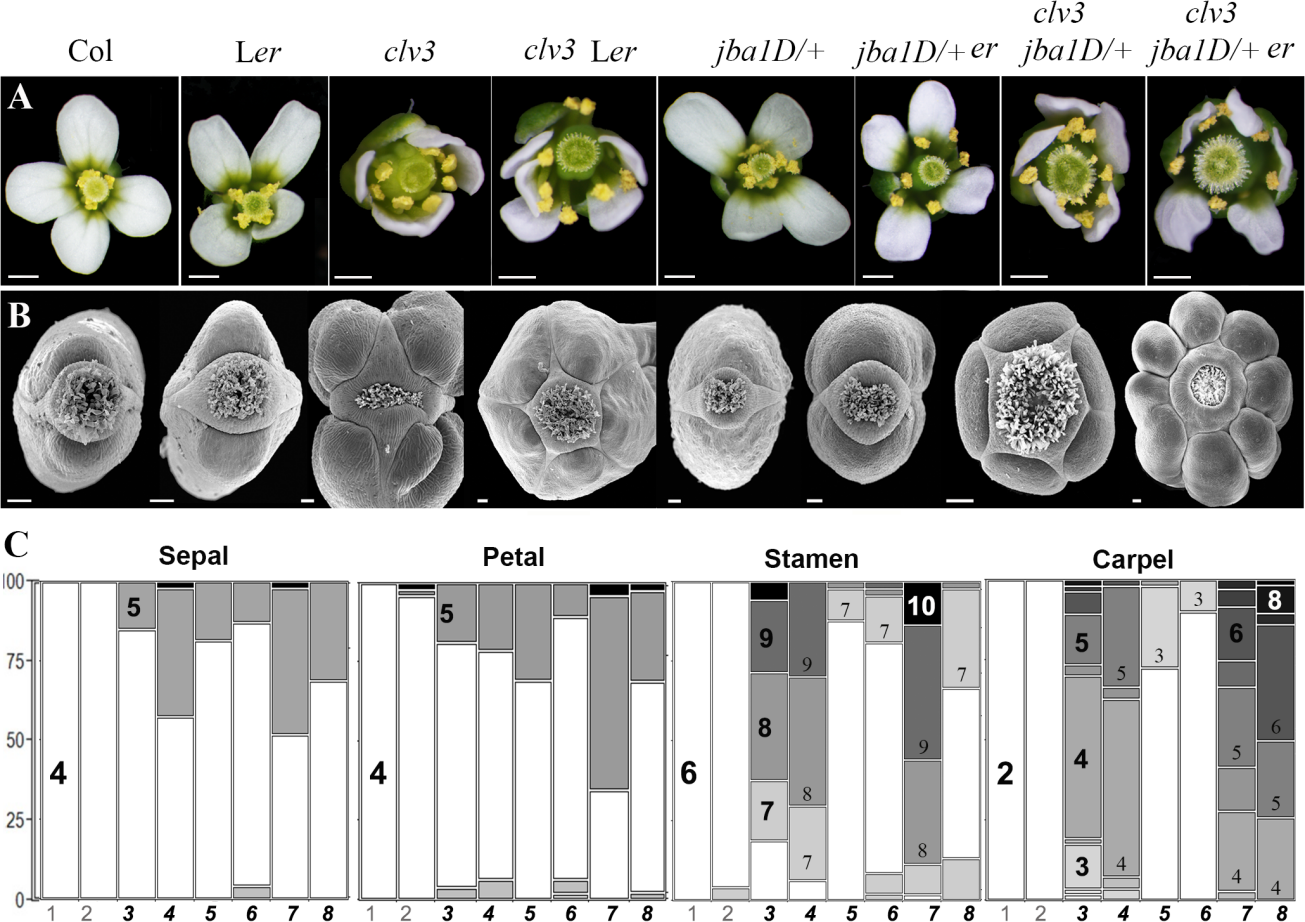
The *ERECTA*, *CLAVATA* and class *III HD-ZIP* pathways display synergistic interaction in regulating floral organ numbers. [**A,B**] Phenotypes of *ER* mutants, *clv3-2* and *jba 1D/+*, single, double and triple mutants. Top views of [**A**] flowers under optical binocular microscope and [**B**] fruits by scanning electron microscopy, from single double and triple mutant plants. Combinations of double and triple mutants with *clv3-2* display increase in numbers of all floral organs (**A**) and extra carpels (**B**) [4 carpels in *clv3-2* (Col), 5 in *clv3-2* (L*er*) and *clv3-2 jba 1D/+* and 8 in *clv3-2 jba 1D/+ er-20*]. Scale bars: Panel A- 500μM and B- 100μM [**C**] Floral organ and primary carpel number are altered in single, double and triple mutants. Numbers of organs appear in the colored code BAR. Genotypes are marked from 1–8 as follow: **1**. Col. **2**. L*er*. **3**. *clv3-2* (Col), **4**. *clv3-2* (L*er*). **5**. *jba 1D/+* **6**. *jba 1D/+ er-20*. **7**. *clv3-2 jba 1D/+* and **8**. *clv3-2 jba 1D/+ er-20*. Data are shown in percentages. N = 45. In all double and triple combinations the numbers of flowers with supernumerary organ compared with their background [Col or L***er***] are highly significant. For each line the significance of the categorical partition of organs number was calculated using Fisher`s exact test (p < 0.05) in comparison to its background (i.e Col or L*er*). The P values are listed in S2 Table.

Analyzing the flowers of the single double and triple mutant combinations we observed two major categories of phenotypes. The first is increase in floral organ numbers, [Fig 1], extra whorls S1 Fig and fasciated FM, all which can be attributed to an enlargement of the FM. To date, in all identified mutants that display enlarged FM but no reduction in floral organ numbers, the increase in meristem size was the results of *WUS* up-regulation [10,30–32,35]. Therefore the first category of phenotype is likely to be a result of *WUS* up-regulation or an expansion of the *WUS* expression domain.

**Fig 2:**
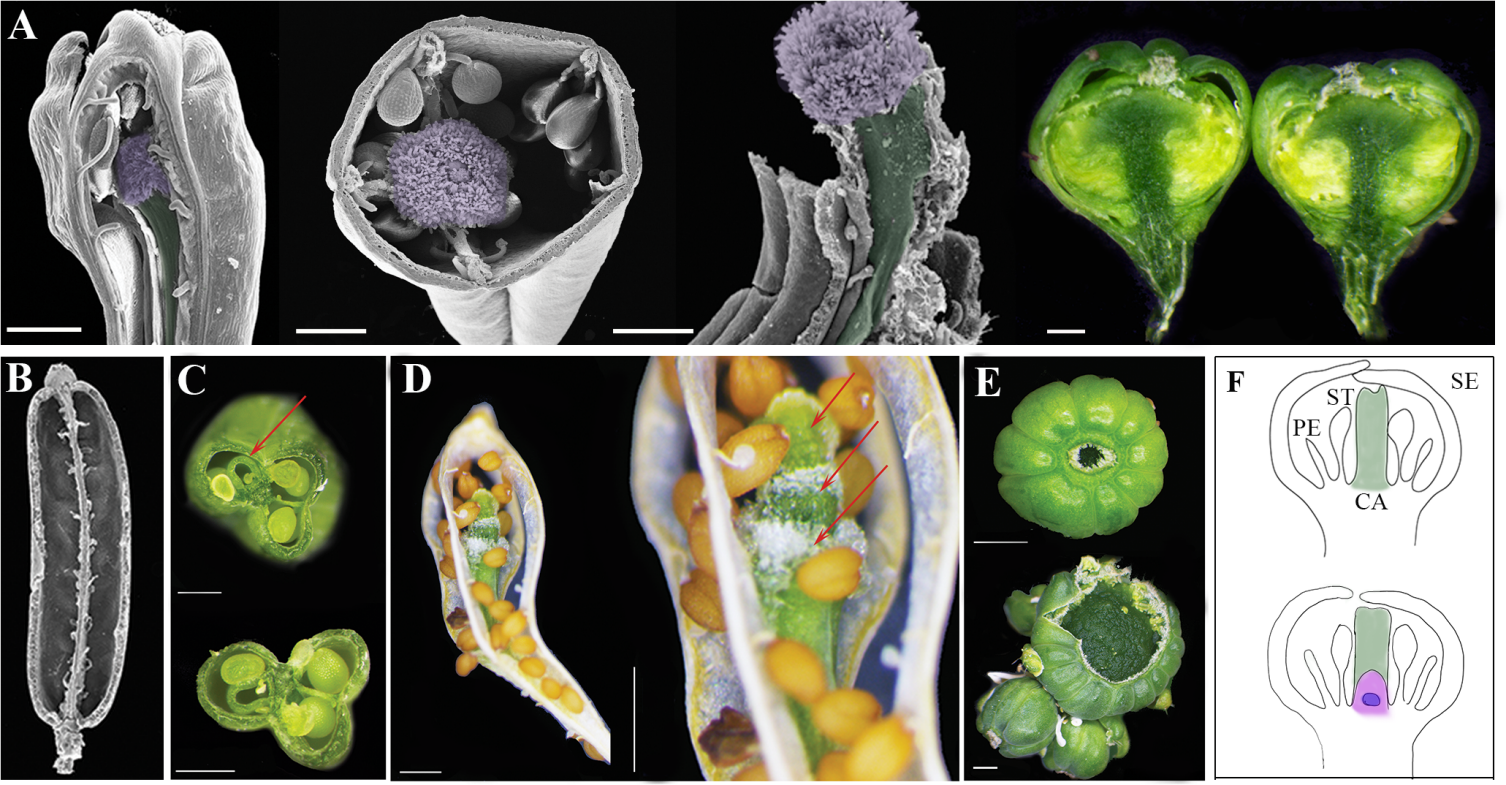
Siliques from plants of *clv3-2* single, double and triple mutants exhibiting extra whorl. [**A**] Extra whorl of carpels develops inside primary carpels of double and triple mutants. SEM views of dissected fruit revealing the fifth whorl of carpels growing from inside the initial carpels [from left to right: *clv3-2* (Col), *clv3-2* (L*er*), and *clv3-2 jba 1D/+*]. Stigma and carpel are false-colored with purple and green, respectively. Dissected *clv3-2 jba 1D/+ er-20* fruit reveals an indeterminate floral meristem (dark green) producing numerous whorls of carpels within carpels. [**B**] SEM views of dissected *jba 1D/+ er-20* fruit showing no extra whorl. [**C**] Dissected *clv3-2* (Col) silique shows extra carpel developed within a primary carpel [red arrow]. [**D**] Dry *clv3-2* (L*er*) silique revealing 3 extra whorls of carpels—fifth, sixth and seventh growing inside each other [red arrows]. [**E**] Top: *clv3-2 jba 1D/+ er-20* sphere-shaped siliques with 8 fused carpels. Lower: later in development the silique displays numerous carpels and a gigantic meristem erupting through the center of the gynoecium indicating a permanent loss of determinacy. Scale bars: Panel A, C and D 500μM, E 1 mm. [F] Arabidopsis flower at stage 6. Top: in wild type flower as the gynoecium no meristem is visible. Lower: In *clv3-2* mutants gynoecium develop while flower meristem is still active (in purple) and *WUS* is still expressed (blue) [14].

**Fig 3:**
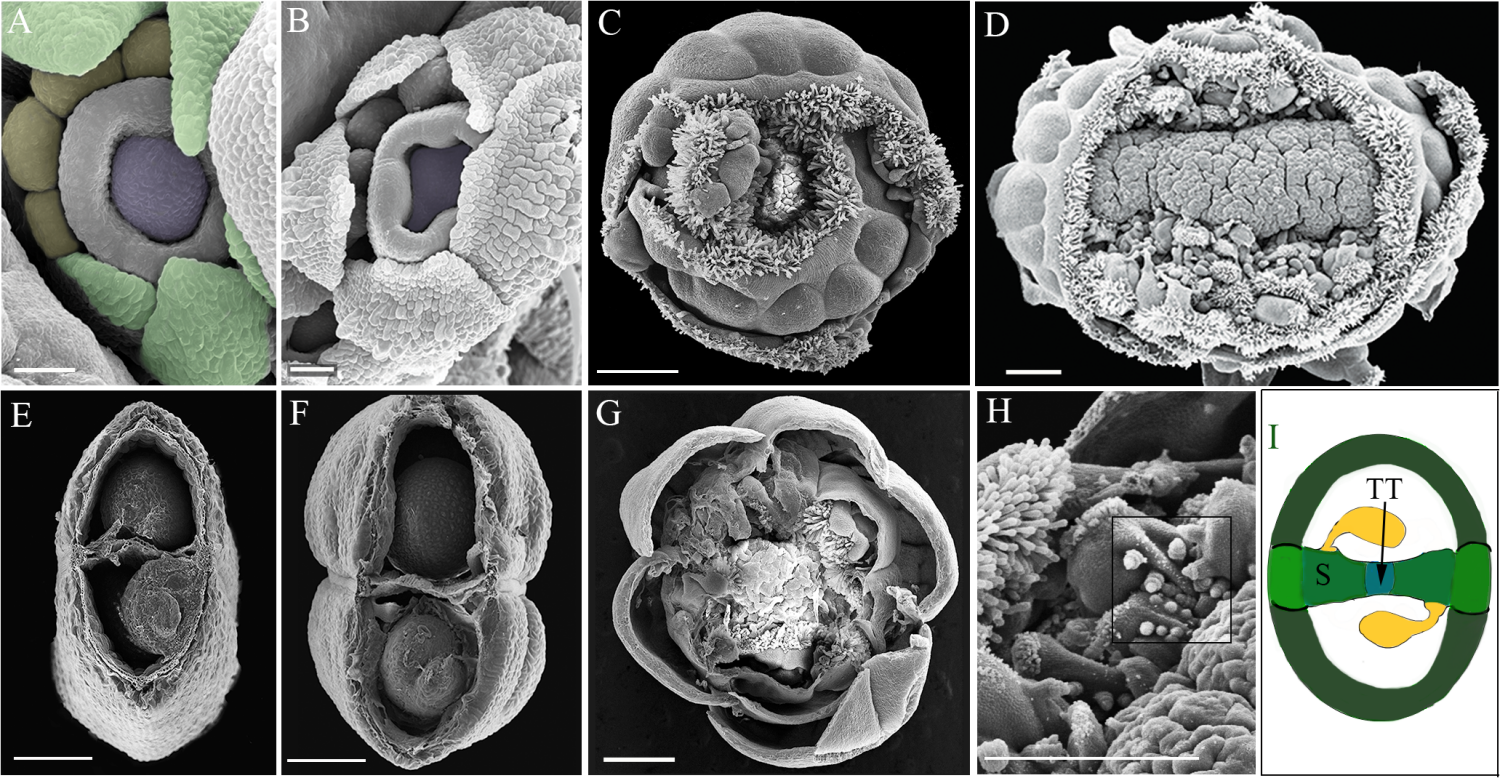
Coordinated FM termination and carpel formation ensure successful reproduction. [A-D] SEM analysis of *jba-1D/+er-20 clv3-2* flower and silique [**A**] flower at stage 7 showing the gynoecial tube with meristem at the center [false-colored with purple]. **[B]** The active meristem blocks the medial ridge growth and septum formation. [**C**] gynoecia exhibits multiple whorls of multi-fused carpels within carpels. [**D**] extremely fascinated meristem encircled by multiple whorls of ectopic fused carpels. [**E – G]** SEM of transverse dissected siliques. [**E and F**] Col and *jba-1D/+er-20* respectively display two fused carpels, septum and developing seed. [**G**] In *jba-1D/+er-20 clv3-*2 the space inside the gynoecium is filled with meristem and ectopic carpels and exhibits no septum. [**H**] In *jba-1D/+er-20 clv3-*2 developing ovule primordia projecting on the placenta are visible (highlighted with a square). [**I**] Transverse section through WT ovary showing the septum [S] stretching from one replum to another, placenta, ovules, and transmitting tract [TT] Krizek, BA [50]. Scale bars: A and B 25μM C, D G and H 500 μM E and F 250 μM.

The second category is supernumerary whorls [Figs 2 and 3] and a total loss of determinacy [Figs 2E and 3D] that can be attributed to extended meristem activity. As was shown previously for mutants such as *clv3*, *ag* and *ult1*, this phenotype of extended meristem activity is most likely the outcome of prolonged *WUS* expression [10,17].

In the single or combined mutants studied, all floral organs were present in higher numbers [Fig 1C], indicating that the increase in organ and whorls numbers result from either an increase in the stem cell population or from the temporal alteration in stem cell termination rather than an homeotic conversion of one organ type into another. An enlarged stem cell population provides more cells for organogenesis allowing more organ primordia to develop.

Among the single mutants, *clv3-2* displayed the most dramatic increase in floral organ number, particularly for stamens and carpels with more than 80% of flowers producing at least one extra stamen and up to a total of 10 stamens, and all flowers producing at least one extra primary carpel [Fig 1]. Furthermore, we observed two types of supernumerary carpels within the gynoecium of the *clv3-2* flowers: one that developed from extended meristem activity, seen as ectopic carpel inside the primary carpel [Fig 2A and 2F], and the other arising from the septum [Fig 2C]. We also observed abnormally wide repla S1 Fig, which might be due to enhanced carpel margin meristem activity [36]. Clark et al [5] showed that the *clv3-2* FMs are significantly taller and only slightly wider than wild-type FMs. This may explain the much greater increase in numbers of stamens and carpels arising from the inner pair of whorls as compared to numbers of sepals and petals arising from the perianth whorl, the outermost two whorls. In the *jba-1D/+* single mutant low percentage of flowers exhibits increased floral organ number although these flowers occasionally displayed petals arising on consecutive whorls, seen as a petal on top of another petal S1 Fig, suggesting that in *jba-1D/+* the FM expands laterally thus providing more cells for the development of extra perianth whorl. Both the L*er* background and *jba-1D/+* enhanced the *clv3-2* flower phenotypes of floral organ numbers [Fig 1] and extra whorl numbers [Fig 2D], displaying up to 3 extra whorls of carpels formed within carpels. However, the enhancement of *clv3-2* by *jba-1D/+* was stronger than the enhancement by L*er*. Indeed, *clv3-2 jba-1D/+* double mutants showed increased numbers of floral organ in all whorls [Fig 1C], with an average of 4.5 sepals, 4.7 petals, 8.5 stamen and 5 carpels [S1 Table]. This increase suggests that the FMs of the *clv3-2 jba-1D/+* double mutant expanded both laterally and horizontally. Among *clv3-2 jba-1D/+* flowers, 46% and 61% developed 5 sepals and 5 petals, respectively, indicating that the FM is already enlarged at early stages of flower development, since sepal primordia arise at flower stage 3, shortly after the emergence of the flower primordium [13]. Altogether, these results suggest that defects in the *CLV3* and *HD-ZIPIII* pathways result in strong up-regulation of *WUS*, starting at the onset of its expression and leading to a born enlarged FM.

Of the three double-mutant combinations studied, *jba-1D/+ er-20* showed the lowest percentage of flowers with supernumerary organs [Fig 1]. Moreover, this double mutant did not produce extra carpel whorls [Fig 2B]. Our analysis showed that in the combinations carrying mutation in *ER*, the carpels and siliques were shorter and wider S2 Fig, as has been previously reported for *er* mutants [37,38]. In wild type flower the gynoecium develops from two congenitally fused carpels that arise from the center of the meristem as a tube [14,15]. Therefore the wide carpels in *er* backgrounds S2 Fig might be due to an increase in the width of the FM central zone resulting in a wider gynoecial tube. Uchida *et al*. [28] showed that loss of function of all three *ER* family genes leads to lateral expansion of the *WUS* expression domain, resulting in flattened and broadened SAMs. A lateral expansion of the *WUS* domain was also shown in *jba-1D/+* seedling SAM [25]. We propose that reduction in both ER and HD-ZIPIII activity in the FM leads to lateral expansion of the *WUS* domain, resulting in spatially alteration of the meristem so that the stem cell population expands horizontally at the expense of a reduced peripheral zone. As a consequence more cells remain as stem cells with strong pluripotent signals that are not competent to differentiate and to be recruited for primordia initiation, leaving fewer cells for the periphery. This can provide an explanation for the low percentage of flowers with supernumerary floral organs that were observed in *jba-1D/+ er-20* plants. The evidence that introducing *er-20* to *clv3-2 jba-1D/+* suppresses the increased number of sepals, petals and stamens phenotype observed in *clv3-2 jba-1D/+* but not the supernumerary carpel phenotype [S1 Table], further support this idea that *er-20* changes the meristem structure, leading to a reduced peripheral zone from where sepals petals and stamens are formed. In the *clv3-2 jba-1D/+ er-20* the most dramatic increase was detected in the number of primary carpels in all the flowers [Fig 1]. We found flowers with up to 14 carpels, all of which developed from the primary gynoecial tube [data not shown]. Our findings suggest that in the triple mutant compared with WT all the FM domains are expanded, providing more cells for all whorls. In addition, the triple mutant flowers exhibited reiteration of multi-fused carpel within carpels that encircle a gradually growing everlasting meristem [Fig 3C] until it bursts and becomes a gigantic meristem [Figs 2E and 3D]. Introducing *wus-1* into the triple mutant background resulted in a *wus-1* phenotype, indicating that *wus* is fully epistatic to the three pathways [data not shown], thus in agreement with previously reported data [25,33].

These strong synergistic phenotypes observed in the triple mutant of extra flower number, supernumerary whorls, total loss of determinacy and extreme enlargement of the meristem as compared to any double mutant combination, indicate that the *CLV3*, *ER* and *HD-ZIPIII* pathways regulate FM activity in parallel.

### Coordinated FM termination and carpel formation ensure successful reproduction

The flowers of the triple mutant continued to produce carpels while stem cells were still proliferating. This provides additional evidence that carpel identity specification and floral stem cell termination are two separable processes [39]. Yet scanning electron microscope analysis of the triple mutant flower at stage 7, which reveals a dome shaped meristem at the center of the gynoecial tube [Fig 3A], strongly emphasizes the fundamental requirement for coordination between those two independent processes to ensure successful reproduction of plants [2,40–42].

In wild-type flowers, following the initiation and growth of the gynoecial tube, the medial ridges meet and fuse to form the septum, which divides the fruit, stretching from the inner side of one replum to the other replum while the ovule primordia arise from the placenta [Fig 3I]. Later, cells in the middle of the septum will form the transmitting tract, a specialized tissue essential for pollen tube growth [15].

In Col, L*er*, *jba-1D/+* and *jba-1D/+ er-20* double mutant, the gynoecium internal tissues developed properly, leading to successful fertilization and seed production [Fig 3E and 3F]. In *clv3-2* single and double mutants, the septum and the transmitting tract developed normally, and fertilization occurred prior to the development of the supernumerary carpel whorls that later disrupted the septum [Fig 2A and 2D]. In contrast, the triple mutant FM showed strong meristematic activity and loss of determinacy. Upon the rising of the gynoecial tube, the active meristem physically blocked the development of the septum and consequently the formation of the transmitting tract [Fig 3A and 3B]. Dissecting mature fruits, we found dense meristematic tissue filling the inner spaces of the gynoecium [Fig 2A]. Though at later stages, when the meristem erupts, some ovules can be seen developing on the exposed placenta [Fig 3H]; with no transmitting tract the pollen tube is incapable of reaching the ovules thus resulting in infertile silique.

We conclude that, although carpel specification and floral stem cell termination are separable processes, both processes must be strictly coordinated for successful reproduction.

### The robust activity of the FM causes FM indeterminacy

Many regulators governing the temporal repression of *WUS* to terminate floral stem cell proliferation are part of the *AG* network, acting as *AG* activators or downstream to AG [2,19,43–47]. AG has recently been shown to directly repress *WUS* expression by binding to the *WUS* locus and recruiting the Polycomb Group (PcG) factor CURLY LEAF for chromatin silencing via deposition of Histone3 Lysine27 repressive methylation marks at *WUS* [19].

Although a decrease in the activity of *CLV3*, *ERECTA* and *HD-ZIPIII* results in loss of determinacy, we propose that these pathways are genetically separable from the *AG* core mechanism of meristem termination: The *AG* gene is required for carpel development as demonstrated by the *ag* mutant having no carpels. Also, we have previously shown that *AG* is required to confer the *jba-1D/+er-20* ectopic carpel phenotype [33]. Since the *jba-1D/+ er-20 clv3-2* triple mutant flowers continually producing carpels it is likely that the *AG* gene is highly expressed and that AG did not lose its function, however fails to repress *WUS*.

We hypothesize that, due to a reduction in these three pathways that restricts *WUS* expression, the *WUS* locus is fully occupied by the transcriptional machinery complexes, keeping it in an active chromatin state and preventing the AG-dependent chromatin modifier mechanism to act. The result would be a failure in shutting down *WUS* and a total loss of FM determinacy. A future challenge will be to test the molecular basis of this hypothetical scenario.

In summary, the analysis of flowers from combined mutants demonstrates that CLV3, ER and HD-ZIPIII participate to regulate FM activity, presumably through distinct pathways, all controlling *WUS* expression. We show that meristem activity termination is not a prerequisite for carpel formation but that coupling FM termination with carpel formation is crucial for seed formation. We also demonstrate that regulation of meristem size is one mechanism that leads to determine flower organogenesis.

## Materials and Methods

### Growth conditions and plant materials

Plants were grown on soil under long days (16 hours light/8 hours dark) and temperatures of 18–22°C. The plant materials used in this study were: Columbia (Col-0), Landsberg *erecta* (L*er*), *jba-1D* [25],*clv3-2* [7], *er-20* [33], *ag-1* (SALK_014999) and *wus-1* [1], ordered from ABRC). Double mutants were generated by crossing *jba-1D/+* plants to *clv3-2* and *er-*20 plants and by crossing *jba-1D/+er-20* plants to *clv3-2* plants.

### Microscopy

Plant images were captured using an Olympus SZX7 Stereomicroscope.

Scanning electron microscopy was performed by fixing tissue in methanol as described [48] and using a JEOL 5410 LV scanning electron microscope.

### Floral organ counting and Statistical analysis

Floral organ counting was performed as described by Fiume et al. [49]. Statistical analysis was carried out using JMP 10 software (SAS Institute Inc., Cary, NC). For each line the significance of the categorical partition of organs number was calculated using Fisher`s exact test (p < 0.05) in comparison to it background (i.e Col or L*er*).

## Supporting Information

S1 Fig Flower and fruit phenotype of the different mutants. [**A**] *jba 1D/+* flower exhibits extra petals and extra whorl of petals. The petal emerged in sequential whorls [red arrow]. [**B**] The *clv3-2* (Col) fruit shows abnormal replum development (dark green tissue). [**C, D**] SEM micrograph of Col (C) and *clv3-2* (Col) fruits (D). The *clv3-2* (Col) fruit shows wider replum in one out of the five repla. [**E**] *clv3-2 jba 1D/+* fruit. Counting the carpels, we assigned a 0.5 value to partial valves, a phenotype observed in all *clv3* double mutants but in high frequently in *clv3-2 jba 1D/+* [replum is highlighted by yellow line]. [**F**] *clv3-2 jba 1D/+ er-20* exhibits sphere-shaped fruit with variable valve size [replum is highlighted]. Scale bars: A -1 mm, B E and F -500μM, C and D -100 μM.
(TIF)

S2 Fig Siliques from single double and triple mutants of *er-20 clv3-2*, and *jba 1D/+*. [**A,B**] typical silique from a wild-type Columbia (Col) (A) and Landsberg erecta (L*er*) (B) is composed of two fused carpels [**C**] *clv3-2* (Col) and [**D**]), *clv3-2* (L*er*), shows siliques comprised of four to five fused carpels. [**E**] *jba 1D/+*[F] *jba 1D/+ er-20* [G] *clv3-2 jba 1D/+*exhibits club-shaped siliques comprised of four to six fused carpels. [**H**] *clv3-2 jba 1D/+ er-20* exhibits short sphere-shaped siliques with numerous extra carpels. Note that siliques from mutants carrying mutation in *ERECTA* are much shorter and border compared to the background plants. Scale bar: 2mm
(TIF)

S1 Table Average of floral organ numbers. Numbers of sepal petal stamen and carpel were counted according to Fiume [49] (number of flowers counted in parentheses) and the mean was calaculated [in bold]. The Std Err Mean appears on the right column.
(DOCX)

S2 Table P Value for Fisher's Exact test presented in Fig. **1.** Number of flower organs of each genotype was compared to the corresponding genotype. The corresponding background on the left compared with mutant on the right [i.e. Col X *jba1D+* compared to Col].
(XLSX)
