# Supplementary figures and images for: The ERECTA, CLAVATA and class III HD-ZIP Pathways Display Synergistic Interactions in Regulating Floral Meristem Activities
Source: PLoS One. 2015 May 6;10(5):e0125408. doi: 10.1371/journal.pone.0125408 (PMC4422654; doi:10.1371/journal.pone.0125408)

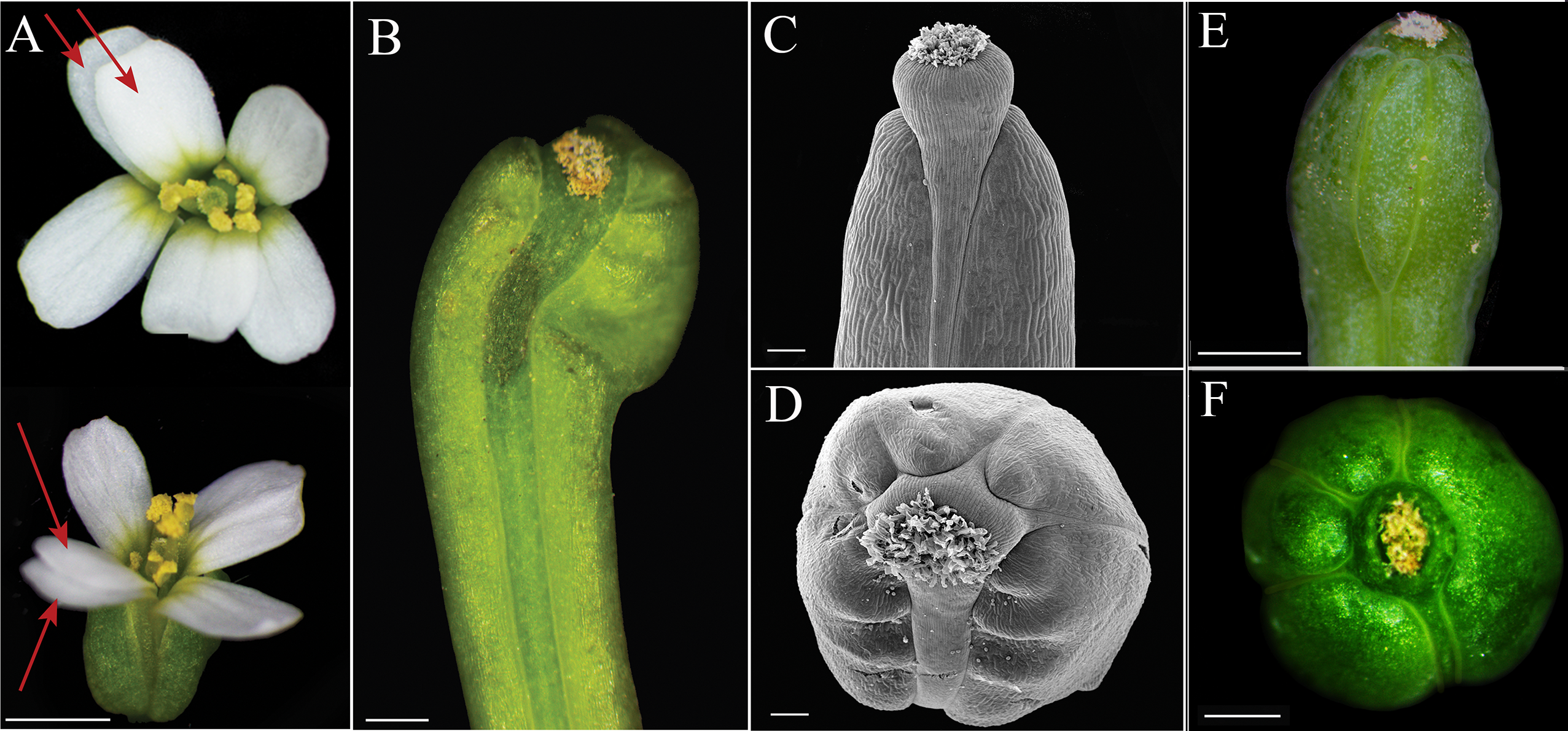

Supplement: S1 Fig — [A] jba 1D/+ flower exhibits extra petals and extra whorl of petals. The petal emerged in sequential whorls [red arrow]. [B] The clv3-2 (Col) fruit shows abnormal replum development (dark green tissue). [C, D] SEM micrograph of Col (C) and clv3-2 (Col) fruits (D). The clv3-2 (Col) fruit shows wider replum in one out of the five repla. [E] clv3-2 jba 1D/+ fruit. Counting the carpels, we assigned a 0.5 value to partial valves, a phenotype observed in all clv3 double mutants but in high frequently in clv3-2 jba 1D/+ [replum is highlighted by yellow line]. [F] clv3-2 jba 1D/+ er-20 exhibits sphere-shaped fruit with variable valve size [replum is highlighted]. Scale bars: A -1 mm, B E and F -500μM, C and D -100 μM. (TIF) [file pone.0125408.s001.tif]

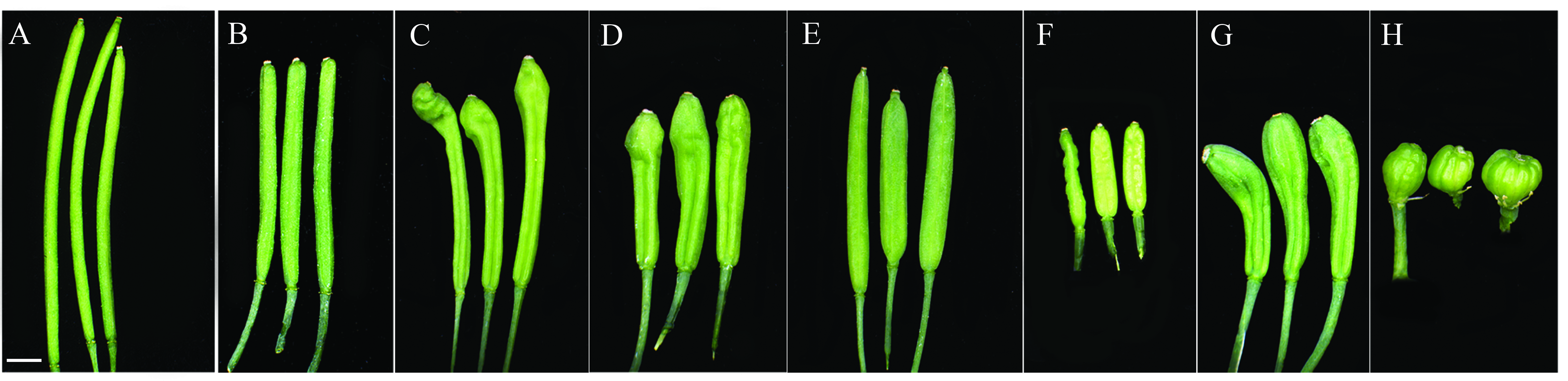

Supplement: S2 Fig — [A,B] typical silique from a wild-type Columbia (Col) (A) and Landsberg erecta (Ler) (B) is composed of two fused carpels [C] clv3-2 (Col) and [D]), clv3-2 (Ler), shows siliques comprised of four to five fused carpels. [E] jba 1D/+[F] jba 1D/+ er-20 [G] clv3-2 jba 1D/+exhibits club-shaped siliques comprised of four to six fused carpels. [H] clv3-2 jba 1D/+ er-20 exhibits short sphere-shaped siliques with numerous extra carpels. Note that siliques from mutants carrying mutation in ERECTA are much shorter and border compared to the background plants. Scale bar: 2mm (TIF) [file pone.0125408.s002.tif]
