# Supplementary material for: The ERECTA, CLAVATA and class III HD-ZIP Pathways Display Synergistic Interactions in Regulating Floral Meristem Activities
Source: PLoS One. 2015 May 6;10(5):e0125408. doi: 10.1371/journal.pone.0125408 (PMC4422654; doi:10.1371/journal.pone.0125408)
Supplement: S1 Table — Numbers of sepal petal stamen and carpel were counted according to Fiume [49] (number of flowers counted in parentheses) and the mean was calaculated [in bold]. The Std Err Mean appears on the right column. (DOCX) [file pone.0125408.s003.docx]

**Supporting Information S1 Table:** Average of floral organ numbers

| **Genotype** Sepal Number Petal Number Stamen Number Carpel Number | | | | | | | | |
| --- | --- | --- | --- | --- | --- | --- | --- | --- |
| **Col** | **4.0** (48) | 0.000 | **4.0** (48) | 0.000 | **6.0** (48) | 0.000 | **2.0** (52) | 0.000 |
| **L*er*** | **4.0** (49) | 0.000 | **4.1** (49) | 0.045 | **6** (49) | 0.029 | **2.0** (53) | 0.000 |
| ***clv3-2* (col)** | **4.2** (53) | 0.050 | **4.2** (53) | 0.063 | **7.8** (53) | 0.161 | **4.2** (57) | 0.137 |
| ***clv3-2*, L*er*** | **4.4** (48) | 0.079 | **4.1** (47) | 0.074 | **7.9** (47) | 0.130 | **4.3** (54) | 0.081 |
| ***jba1D/+*** | **4.2** (49) | 0.056 | **4.3** (49) | 0.067 | **6.1** (49) | 0.058 | **2.3 (**51) | 0.070 |
| ***jba1D/*+, *clv3-2*** | **4.5** (52) | 0.075 | **4.7** (52) | 0.075 | **8.6** (52) | 0.127 | **5** (42) | 0.148 |
| ***jba1D/+, er-20*** | **4.1** (48) | 0.060 | **4.0** (48) | 0.071 | **6.1** (49) | 0.110 | **2.1** (51) | 0.042 |
| ***jba1D/+, clv3-2, er-20*** | **4.3** (46) | 0.070 | **4.3** (46) | 0.083 | **6.2** (46) | 0.105 | **5.5** (58) | 0.165 |

[Numbers given are the mean, On the column to the right, Std Err Mean. In parentheses,

N, number of flowers counted]
